# Volatile Anesthetics Regulate Anti-Cancer Relevant Signaling

**DOI:** 10.3389/fonc.2021.610514

**Published:** 2021-02-26

**Authors:** Jiaqiang Wang, Chien-shan Cheng, Yan Lu, Shen Sun, Shaoqiang Huang

**Affiliations:** ^1^ Department of Anesthesiology, The Obstetrics and Gynecology Hospital of Fudan University, Shanghai, China; ^2^ Department of Integrative Oncology, Fudan University Shanghai Cancer Center, Shanghai, China; ^3^ Department of Oncology, Shanghai Medical College, Fudan University, Shanghai, China; ^4^ Department of Anesthesiology, Shanghai First Maternity and Infant Hospital, Tongji University School of Medicine, Shanghai, China

**Keywords:** volatile anesthetics, inhalation anesthesia, anti-cancer, volatile anesthesia, mechanism

## Abstract

Volatile anesthetics are widely used inhalation anesthetics in clinical anesthesia. In recent years, the regulation of anti-cancer relevant signaling of volatile anesthetics has drawn the attention of investigators. However, their underlying mechanism remains unclear. This review summarizes the research progress on the regulation of anti-cancer relevant signaling of volatile anesthetics, including sevoflurane, desflurane, xenon, isoflurane, and halothane *in vitro*, *in vivo*, and clinical studies. The present review article aims to provide a general overview of regulation of anti-cancer relevant signaling and explore potential underlying molecular mechanisms of volatile anesthetics. It may promote promising insights of guiding clinical anesthesia procedure and instructing enhance recovery after surgery (ERAS) with latent benefits.

## Introduction

Cancer describes diseases characterized by uncontrolled cell division and tissue invasion. Cancer hallmarks include maintaining proliferation signals, evading cell death, resisting treatment, enabling invasion, inducing angiogenesis, and activating metastasis (1). Cancer treatment strategies include traditional methods, such as surgery, chemo- and radio-therapy; and newer methods such as a ligand or receptor-based target therapy, stem cell therapy, and various forms of novel drug delivery systems (2). Until now, cancer is still an insurmountable problem worldwide, leading to high morbidity and mortality (3).

Volatile anesthetics, including sevoflurane, desflurane, xenon, isoflurane, halothane and others, are used for inhalational anesthesia in clinical practice. Volatile anesthetics target specific central nervous system receptors to perform anesthetic functions, such as the neuronal GABA_A_ receptor, NMDA receptor and glutamate receptor subtypes. Volatile anesthetics can also affect cells by changing transcriptional elements, thereby changing specific characteristics of cell function. Previous studies have shown that volatile anesthetics have organ protection effects (4, 5). Recently, researchers have focused on the regulation of anti-cancer relevant signaling of volatile anesthetics on different kinds of cancer *in vitro*, *in vivo*, and clinical studies, but the specific mechanism remains unclear.

Everything has two sides. Some studies reported that sevoflurane (6–12), isoflurane (7, 10, 11) and halothane (13–15) may play tumor-promoting effects. We compared the articles and found that the controversy may come from different cancer types, cell lines, incubation concentrations and other conditions. More research on differences should be studied to discover potential conditions for volatile anesthetics and suitable cancer types. Analysis articles aimed at comparing opposite findings are also welcome. More *in vivo* and clinical studies should be conducted to further determine the regulation of anti-cancer relevant signaling of volatile anesthetics to guide clinical anesthesia procedures.

This review summarized the regulation of anti-cancer relevant signaling, including anti-proliferation, anti-migration and invasion, anti-metastasis, apoptosis-inducing effects, and the underlying mechanisms of volatile anesthetics. It may be instructive for future clinical inhalation anesthesia and beneficial for ERAS.

## Regulation of Anti-Cancer Relevant Signaling

### Sevoflurane

Sevoflurane (C_4_H_3_F_7_O, **Table 1**) is one of the most commonly used volatile anesthetics. It is a colorless and sweet-smelling inhalation anesthetic used to induce and maintain general anesthesia. For induction and maintenance of general anesthesia, sevoflurane concentration ranges from 0.5%–5% and less than 4%, respectively. In the electrophysiological study of neurons and recombinant receptors, sevoflurane is a positive allosteric modulator of the GABA_A_ receptors (16–18). However, it can also act as a NMDA receptor antagonist (19), enhancing glycine receptor electro-currents (20) and inhibiting nAChR (21) and 5-HT3 receptor currents (22, 23). Sevoflurane is particularly non-irritating to the respiratory tract, so it is particularly suitable for asthma patients’ anesthesia.

**Table 1:** The volatile anesthetics.

Volatile Anesthetics	Sevoflurane	Desflurane	Xeon	Isoflurane	Halothane
Chemical Formula	C_4_H_3_F_7_O	C_3_H_2_F_6_O	Xe	C_3_H_2_ClF_5_O	C_2_HBrClF_3_
CAS ID	28523-86-6	57041-67-5	20222-53-1	26675-46-7	151-67-7
Molecular Formula	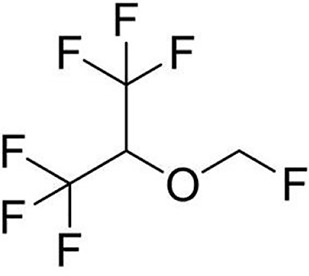	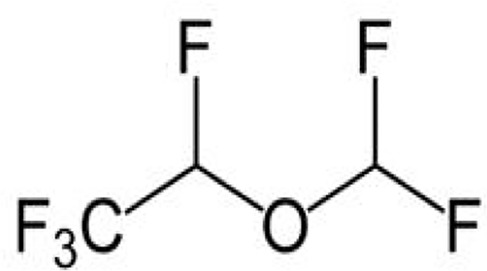	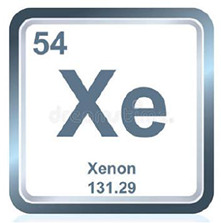	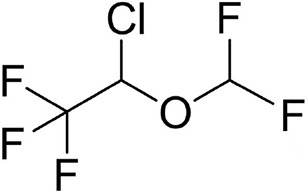	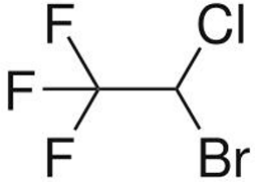

## Sevoflurane and miRNAs

miRNAs are single-stranded, highly conserved small non-coding RNAs. More and more studies have shown that miRNAs affect cancer proliferation, metastasis, and invasion. In addition, miRNA expression can also determine the pathogenesis, diagnosis, and diseases prognosis of cancer (24). Recently, miRNAs are proposed to function both as oncogenes and tumor suppressors by regulating various target gene expressions (25, 26). Oncogenes are genes that may cause cancer and are often mutated and expressed at high cancer cell levels. On the other hand, tumor suppressor genes, or anti-oncogenes, are genes that protect cells from malignant transformation (27). Recent studies have found that Sevoflurane can regulate miRNA expression.

miR-203 has been implicated to play an essential role cancer proliferation regulation and are of potential diagnostic value. It is reported that miR-203 can act both as an oncogene and tumor suppressor gene in the development of different types of cancers (28–30). Fan and colleagues treated colorectal cancer cells (CRC, cell lines: SW620 and HCT116) with 1%, 2%, and 4% sevoflurane for 6 h to investigate the regulation of anti-cancer signaling of sevoflurane in CRC cell lines. The study demonstrated a proliferation suppression effect of sevoflurane, along with its migration and invasion inhibitory effects, by regulating the ERK/MMP-9 pathway through miR-203/Robo1 (31). In another study, MDA-MB-231 and MCF-7 breast cancer cells were exposed to 2% sevoflurane for 6 h. Results demonstrated that clinical concentration of sevoflurane could significantly suppress the proliferation of breast cancer cells *via* up-regulation of miR-203 (32).

miRNA-637 is as a tumor suppressor effect and plays crucial role in carcinogenesis and cancer progression (33–35). Emerging evidence suggested that miRNA-637 regulates the migration and invasion of glioma cells (36). In glioma *in vitro* models, U251 cells were treated with sevoflurane (1.7%, 3.4%, 5.1%) for 6 h. Yi and colleagues reported that sevoflurane inhibited glioma migration and invasion by up-regulating miRNA-637 and suppression of downstream Akt1 expression and activity (37).

In lung cancer, the regulation of anti-cancer signaling of sevoflurane by regulating miRNA has also been investigated (38). A study aimed at elucidating sevoflurane’s effect on the miRNA in lung cancer cells showed that A549 cells pretreated with 3% sevoflurane for 0.5 h caused an increase in apoptosis, thereby significantly reduced the risk of cancer cell metastasis and improving patients’ postoperative survival rate. Sevoflurane pretreatment up-regulated tumor suppressor miRNA-21, miRNA-221 and down-regulated oncogenic miRNA-34a in A549 cancer cells (39).

miR-124 is widely expressed in the nervous system (40). Rho-associated coiled-coil–containing protein kinase (ROCK1) plays essential roles in regulating tumorigenesis, cell apoptosis, invasion and migration (41). As reported by Cao et al., 4.1% sevoflurane pretreatment for 4 h inhibits glioma proliferation, invasion, and metastasis in U251and U87 cells through enhancing miR-124-3p level, thereby suppressed tumor malignancy-related ROCK1 signaling pathway (42).

## Sevoflurane and MMPs

Matrix metalloproteinases (MMPs) are proteolytic enzymes that contribute to the degradation of extracellular matrix and basement membrane and are associated with cancer cell invasion. Among them, MMP-2 and MMP-9 are remarkably up-regulated in malignant tumors and contribute to cancer invasion (43).

Compared with normal brain tissue, MMP-2 is highly expressed in gliomas. MMP-2 has shown multiple effects in tumor progression, and promoted glioma malignancies (44, 45). Research by Hurmath et al. suggested that 2.5% sevoflurane incubation for 1.5 h suppressed the migration capability of U87MG glioma cells by down-regulation MMP-2 activity (46).

Degrading extracellular matrix is considered first step of tumor cell progression. Prior to tumor invasion into blood vessels or lymph nodes, tumor cells degrade the extracellular matrix, such as MMP-9 (47). Research evidence showed that in an *in vitro* reperfusion injury model, preconditioning with 2.2% sevoflurane for 45 min reduced MMP-9 release from human neutrophils by interfering with its downstream CXCR2 and its upstream PKC. By down-regulating MMP-9, sevoflurane suppressed MC-38 colon cancer cells migration (48).

Sevoflurane also demonstrated growth and invasion inhibitory effects in lung adenocarcinoma A549 cell line (49). The mechanism of its growth inhibition may be related to the synergistic down-regulation of X-linked inhibitor of apoptosis (XIAP) and survivin. Furthermore, its the synergistic effect of invasion inhibition may be related to the down-regulation of MMP-2 and MMP-9.

## Sevoflurane and Cell Cycle

Abnormal cell proliferation is most related to the influence of cell cycle regulation (50). 2.5% Sevoflurane treatment for 4 h significantly inhibited A549 cells’ proliferation, invasion and induce cellular apoptosis and arrest the cell cycle at the G2/M phase (51). Furthermore, 2% sevoflurane preconditioning for 6 h possessed anti-proliferative and pro-apoptotic effects, possibly related to the down-regulation of XIAP and survivin expression and caspase-3 activation. Cell cycle arrest in the G2/M phase is associated with the down-regulation of cyclin A, cyclin B1, and cdc2 kinase expression. Sevoflurane can significantly inhibit breast cancer cells’ proliferation by blocking the cell cycle in the G1 phase (32).

## Sevoflurane and Cell Apoptosis

In colonic cancer, 3% sevoflurane incubation for 1 or 2 h induced late apoptosis in Caco-2 cells *in vitro* (52). The study reported that sevoflurane intervention increases CYP2E1, caspase-3, and p53 expression. Furthermore, sevoflurane also facilitates an early increase of *de novo* ceramide synthesis. These results suggested that sevoflurane acts on both signaling pathways and metabolic pathways *in vitro*.

In neck squamous cell carcinoma (HNSCC) cancer, 2% and 4% sevoflurane pretreatment for 2, 4, 6, and 8 h inhibited proliferation, invasion, migration, and induced cellular apoptosis of FaDu and CAL-27 cell lines (53). The anti-proliferation effect of sevoflurane was associated with the downregulation p-Akt expression, and the cell apoptosis effect was associated HIF-1α activation, which regulated the Fas/FasL signaling pathway.

## Sevoflurane and HIF-1α

The HIF is a family of transcription factors that involved in crucial aspects of cancer biology, such as cell proliferation, angiogenesis, metabolomic adaptation, and metastasis (54). Sevoflurane preconditioning (1.5%, 2.5%, or 3.5% sevoflurane incubation of A549 cells for 4 h) can inhibit the proliferation and invasion of lung cancer A549 cells induced by hypoxia, which may be related to the down-regulation of HIF-1α and its downstream genes XIAP, survivin, fascin, and HPA (55). Under hypoxia conditions, HIF-1α activation is dependent on the activation of the p38 MAPK signaling pathway. Also, the study proved that sevoflurane partially reversed the hypoxia induced p38 MAPK activity.

Activation of HIF-1α by sevoflurane regulates the Fas/FasL signaling pathway to exert cell apoptosis as demonstrated above (43).

## Sevoflurane and VEGF

Hypoxia regulates transcriptional factor HIF-1, which regulates hypoxia-inducible angiogenic factor VEGF (56). VEGF is an important survival factor for endothelial and tumor cells. In tongue squamous cell carcinoma cell (TSCC), SCC-4 cells incubated with 4.1% sevoflurane for 24 and 72 h was shown attenuated VEGF level *via* increasing the DNA methylation of the VEGF promoter region *in vitro (* 37).

## Sevoflurane and Wnt/β-Catenin Signaling

Sevoflurane was found to significantly inhibited the growth of a panel of chronic myeloid leukemia (CML) cell lines (KCL22, K562, KU812, LAMA84 and KBM-7) (57). It also inhibited proliferation, differentiation and self-renewal capacities of CML CD34 cells. Mechanistically, it is purposed that 2%, 4%, or 8% sevoflurane preconditioning for 24 h dose-dependently decreases β-catenin and c-Myc expressions and activities in K562 and CML CD34 cells. The findings also reveal the Wnt/β-catenin pathway may be important targets of volatile anesthetics in cancer treatment.

## Sevoflurane and Platelets Activation

It has been demonstrated that activated platelets contribute to tumor cells’ metastatic ability and protect circulating tumor cells from immune cells (58, 59). Furthermore, surgery stress potentiates platelets activation. Thus, a promising therapeutic strategy of preventing platelets-induced metastasis during cancer surgery procedure is much needed. Previous study suggested that sevoflurane attenuates platelet activation in lung cancer patients by reducing GPIIb/IIIa, CD62P, and PAR levels and these effects are further validated *in vitro*. It is indicated that sevoflurane at 1 MAC reduces platelets-induced invasive potential of lung cancer A549 cells through decreasing platelets activity (60).

### Desflurane

Desflurane (C_3_H_2_F_6_O, **Table 1**) is widely used for anesthesia maintenance in contemporary clinical work. Characterized by low blood solubility, it functions as the fastest in acting and revival of volatile anesthetics. Desflurane is prohibited for anesthesia induction in infants and young children due to its potential of causing adverse reactions.

## Desflurane and MMPs

In an *in vitro* reperfusion injury model, MC-38 colon cancer cells were incubated with 6% of desflurane for 45 min. It was demonstrated that desflurane could reduce the deliverance of MMP-9 by intervening downstream of the CXCR2 pathway. By down-regulating MMP-9, desflurane reduced the degradation of matrigel and the migration of colorectal cancer cells (48).

## Desflurane and DFS

One study conducted a historical cohort study in which all patients received the primary cytoreductive surgery for stage III epithelial ovarian cancer, and the evaluation factor was disease-free survival (DFS). Studies have found that, compared with other volatile anesthetics, desflurane decreased the relative risk of cancer recurrences and is associated with improved DFS after surgery (61).

## Desflurane and the Immune System

According to a randomized trial, during the perioperative period, desflurane anesthesia for breast cancer surgery can induce an adequate immune response in terms of maintaining the ratio of CD4(+)/CD8(+) T cells (62). Regarding leukocytes and NK cells, desflurane anesthesia’s adverse immune response is less than that of propofol.

### Xenon

Xenon (Xe, **Table 1**), is the most stable gas of noble gas, which targets the glycine binding site of the NMDA receptor and the KATP channel. Xenon gas can dissolve in the fat of cells, causing cell anesthesia and swelling, thereby temporarily stopping the function of nerve endings. Owing to that xenon does not increase the sensitivity of myocardium to catecholamines-induced arrhythmia and it inhibits myocardial contraction but with minimal inhibitory effect on cardiovascular function, xenon is suitable for and widely used in cardiovascular surgery.

## Xenon and RANTES

Regulated on activation, normal T cell expressed and secreted (RANTES), also known as CCL5 and functioning on receptor CCR5, is a cytokine that continues to increase in breast cancer subtypes (63) and is associated with promoting breast cancer metastasis and progression (64, 65). Ash et al. investigated the effect of xenon on migration and oncogene expression in human breast adenocarcinoma cells (66). It was demonstrated that 70% xenon incubation for 20 min inhibited the migration of estrogen receptor-positive (MCF-7) and negative (MDA-MB-231) breast cancer cells and reduce the pro-angiogenic factor’s release.

## Xenon and PMCA

It is reported that xenon, at partial pressures ranging from 0.5 to 1.5 atm (equivalent to 0.5 to 1.6 MAC) for 30 min, can inhibit the pumping of plasma membrane Ca^2+^-ATPase (PMCA) in synaptic plasma membrane vesicles in rat C6 glioma cells. This mechanism may inhibit the physiological functions of cancer cells (67).

Owning to the inertness of xenon, it can only be extracted and liquefied, but cannot be synthesized artificially. Therefore, it is costly to utilize xenon. However, with the development of novel manufactured techniques, xenon is gradually adopted in various countries. Although it has not been used clinically, it is suggested that xenon may regulate anti-cancer relevant signaling and is worthy of further exploration.

### Isoflurane

Isoflurane (C_3_H_2_ClF_5_O, **Table 1**) is colorless and of pungent odor. It is used for anesthesia maintenance and has the properties of reducing pain sensitivity and relaxing muscles. Isoflurane may bind to GABA and Glycine receptors, but has different effects. However, the clinical application of isoflurane is gradually replaced by sevoflurane and desflurane due to its potential complication of inducing epileptiform EEG.

## Isoflurane and Glutamate

Glutamate is the primary excitatory neurotransmitter and acts as an effective neurotoxin when overexcited. Therefore, the extracellular glutamate concentration must be kept low to carry out neurotransmission and prevent damage effectively. Isoflurane incubation of C6 glioma cells can increase the expression and activity of type 3 excitatory amino acid transporter (EAAT3) through a pathway that depends on PKC and PI3K, thereby exhibiting higher glutamine in a time- and concentration-dependent manner (68). In addition, it has been reported that potential treatments targeting glutamine metabolism can be used to treat many types of cancer (69).

## Isoflurane and COX-2

Cyclooxygenase (COX), also known as prostaglandin H_2_ (PGH_2_) synthase, is an essential enzyme for converting arachidonic acid to PGH_2_. Studies have shown that inhibition of COX is related to tumor behavior. A research report pointed out that 1.4% isoflurane treatment for 0.5 h significantly reduced the enhancement of COX-2 and the release of PGE2 of human laryngeal papilloma cells. By inhibiting the activation of p38 MAPK, isoflurane inhibited cell proliferation and apoptosis evasion (70).

## Isoflurane and Cell Apoptosis

An *in vitro* and *in vivo* study reported that isoflurane incubation (2 mg/ml, 48 h) could not only inhibited liver cancer growth, but also decreased cell viability in liver cancer patient. The specific mechanism involves upregulating the expression levels of proapoptotic genes (caspase-3 and caspase-8) and downregulating anti-apoptotic (Bcl-2 and Bax) mRNA expression. Furthermore, isoflurane treatment inhibited migration and invasion of hepatic carcinoma cells. The molecular mechanisms underlying the tumor aggressiveness suppressive role of isoflurane involved regulation of NF-κB activity, and the PI3K/AKT signaling pathway (71).

### Halothane

Halothane (C_2_HBrClF_3_, **Table 1**) is liquid anesthetic with colorless, clear, volatile and scented properties. It is unstable in nature and can be slowly decomposed by light and heat. Similar to other volatile anesthetics, halothane performs its anesthetic function by activating GABA-A, glycine, and NMDA receptors (72–74).

## Halothane and Energy Metabolism

In the presence of halothane, glucose uptake and lactate output increase and oxygen consumption is inhibited, 0.9% halothane incubation showed 50% inhibition in the heteroploid strain and 0.35% halothane in the mouse sarcoma I strain. Also, population growth and high-energy phosphate production are diminished. A variety of biochemical mechanisms implicating the mitochondrial mechanisms may be involved (75).

## Halothane and DNA or RNA Synthesis

Jackson et al. reported that halothane treatment varied in 0.1%–5.0% for 24 h was found to inhibit cell multiplication and cell growth in rat hepatoma cells, with 2.5% halothane pretreatment for 6 h being the most significant (76). Another study suggested that 0.1%–5.0% halothane preconditioning for 2 h inhibited the incorporation of extracellular thymidine into DNA, thus inhibiting DNA synthesis on hepatoma HTC cells (77).

Studies on cytotoxicity and anti-proliferative effects indicate that the anti-tumor ability of inhaled anesthetics may be halothane> sevoflurane> isoflurane. In human colon cancer (Caco-2), laryngeal cancer (HEp-2), and poorly differentiated cells from lymph node metastasis of colon carcinoma (SW-620), 1.5% halothane preconditioning for 2, 4, and 6 h showed significantly growth inhibitory effect. Among the cell lines studied, halothane significantly reduced the DNA and RNA synthesis in Caco-2 and Hep-2 cells. Furthermore, decrease in DNA, RNA and protein synthesis were observed in Caco-2 and Hep-2 cells. In SW620 cells, protein synthesis were decreased. A DNA fragmentation was observed in MIA PaCa-2 and Caco-2 cells (78).

## Halothane and Morphology

In a study, cultured neuroblastoma cells were incubated of halothane (100 or 1,000 ppm) for 4, 12, or 24 h *in vitro*. Exposure to halothane resulted in significant changes in the actin distribution pattern of neuroblastoma cells, and the cells exhibited characteristic morphological changes (79).

A clinical study showed that the type of anesthesia influenced the end results of therapy of cancer patients, the survival rates of patients receiving halothane anesthesia were much higher than ether-anaesthetized ones. The mechanism may involve influences of anesthetics on the pituitary-adrenal cortex system and carcinoma development and the role of immunity in tumor cell implantation and growth of metastases (80).

It is reported that 1.5 and 2.21 mM halothane incubation induced genotoxic and cytotoxic effects in lung cancer A549 cells *in vitro*. Consequences of the regulation of anti-cancer relevant effects involved reducing cell viability, inhibiting mitotic activity, and destroying the nucleus and nucleolus structure (81).

Investigations of volatile anesthetics on cellular morphological differentiation unveiled that 0.3%–2.1% halothane preconditioning up to 72 h inhibited neuroblastoma cells (clone NB2a) differentiation, the inhibition of neurite extension dose dependently and virtually abolished microspike formation even at the lowest concentration incubated. The mechanism was demonstrated that halothane inhibited neurite extension and abolished microspike formation (82).

## Halothane and ICAM-1

Intercellular adhesion molecule 1 (ICAM-1) is a cell surface glycoprotein commonly expressed on endothelial cells and immune cells. Recent studied have reported that ICAM-1 is expressed in several tumors, and high expression has been positively correlated with metastatic potential. In human melanoma SK-MEL-37 cells, 4% halothane incubation for 3, 6, 12, or 24 h was demonstrated to perform lower ICAM-1 expression. Thus it was concluded that halothane possessed tumor metastasis inhibiting property by down-regulating ICAM-1 expression *in vitro (* 83).

## Halothane and PMCA

The plasma membrane Ca^2+^-ATPase (PMCA), is a ubiquitously expressed Ca^2+^ pump that releases Ca^2+^ from the calcium reservoir to the cytoplasm, regulating physiological functions including cell movement, growth and differentiation. Halothane, at concentration ranging from 0.5% to 1.75% (0.5 to 1.6 MAC), significantly inhibited plasma membrane vesicles Ca^2+^ uptake dose-dependently in rat C6 glioma cells, B104 neuroblastoma cells and PC12 pheochromocytoma cells (67).

## Discussion

Recent studies have shown that volatile anesthetics regulate of anti-cancer relevant signaling in human cancers. Specifically, exposure to volatile anesthetics can change the biological response of cancer cells or regulate the gene expression of cancer cells, thereby exerting apoptosis induction, anti-invasion, anti-migration and other anti-cancer properties. There are many studies on the regulate of anti-cancer relevant signaling of sevoflurane, but more research on desflurane, which is also commonly used in clinical practice, is needed. As for the promising new type of inhaled anesthesia xenon, due to the difficulty of production and high price, it has not yet been widely used in clinical practice. The prospect of scientific research is worth exploring. There lacks of research articles concerning the regulation of anti-cancer relevant signaling of enflurane, methoxyhalothane, and ether. These documents are no longer in clinical use, so we did not discuss them in this review.

Although volatile anesthetics are not traditionally regarded as anti-cancer drugs, more and more research have focused on the potential anti-cancer properties. Volatile anesthetics mainly act on NMDA and GABA receptors. Although it is still unclear why anesthetics can regulate of anti-cancer relevant signaling, it has been reported that activated receptors can exert regulate anti-cancer-related signaling in cancer cells (84–87). Therefore, studying the regulation of anti-cancer relevant signaling of volatile anesthetics and their related receptors is a new and enlightening insight with important significance, and therefore may make outstanding contributions to cancer biology. Considering different types of cancer have different sensitivity to volatile anesthetics, this current review may guide the choice of volatile anesthetics to best improve the clinical prognosis of cancer patients and improve their postoperative recovery (ERAS).

The shortcomings of contemporary researches relatively lack of animal studies, clinical trials, genomics analysis and big data analysis. Volatile anesthetics exert anesthetic functions *via* passing through the respiratory tracts and blood-brain barrier and then acting on the receptors. Do volatile anesthetics demonstrate exceptional sensitivity of anti-cancer relevant signaling in lung cancer and brain tumor? More specific and compelling trials are needed, especially those related to sevoflurane and desflurane which is clinically widely used, to clarify the relationship between anesthetics and tumor prognosis, and to provide more precise guidance for anesthesia management.

## Conclusion

**Figure 1:**
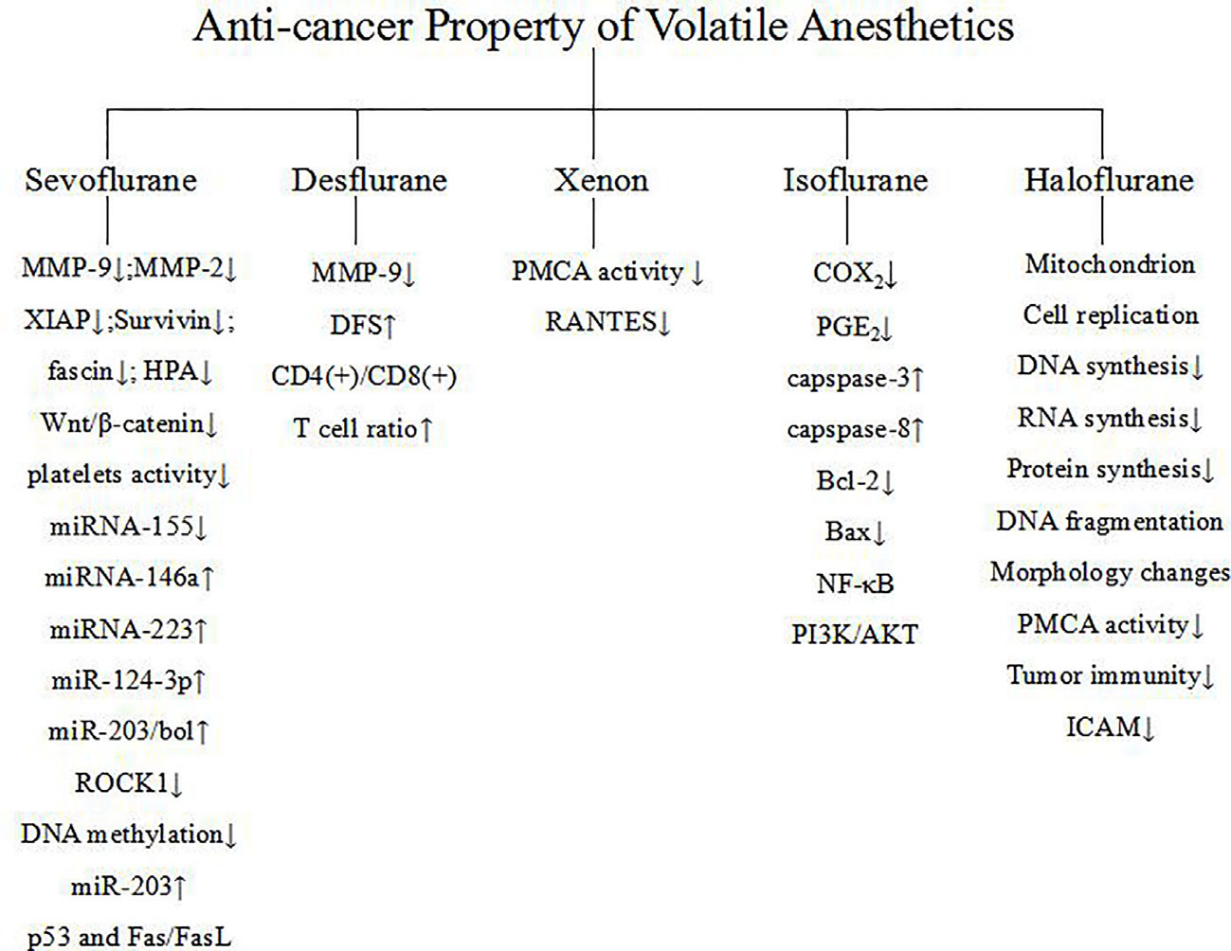
Mechanisms of regulation of anti-cancer relevant signaling of volatile anesthetics.

From the above research and investigation, it can be concluded that volatile anesthetics could regulate anti-cancer relevant signaling (**Table 2**). The underlying mechanism involves miRNA, transcription factors, apoptotic pathway, MMP, etc. **Figure 1**. Although the current research may shortcomings, more in depth studies, especially clinical research, is warranted to clarify the regulation of anti-cancer relevant signaling of volatile anesthetics.

**Table 2:** Regulation of anti-cancer relevant signaling of volatile anesthetics.

Volatile Anesthetics	Cancer Type	Cell Line	Treatment	Effects	Mechanisms	References
Sevoflurane	Colorectal cancer	SW620;HCT116	Concentration:2.5%; Time: 90 min	Inhibition of cell migration and invasion	Addressing ERK/MMP-9 signaling pathway by regulating miR-203/Robo1 expression	
	Glioma	U87MG	Concentration:2.5%; Time: 24 h	Inhibition of cell migration	MMP-2 activity↓	Hurmath FK et al. (46)
	Lung adenocarcinoma	A549	Concentration:2.5%; Time: 24 h	Inhibition of cell growth and invasion	XIAP↓, survivin↓, MMP-2↓, MMP-9↓	Liang H et al. (49)
	CML	KCL22, K562, KU812, LAMA84 and KBM-7	Concentration:2%, 4%, 8%; Time: 24 h	Inhibition of cell growth, proliferation, differentiation and self-renewal capacities	Inhibiting Wnt/β-catenin in a p38 MAPK-independent manner	Ruan XG et al. (57)
	Lung cancer	A549	Concentration: 1MAC	Suppression of platelets-induced invasion	Decreasing platelets activity *via* GPIIb/IIIa, CD62P, and PAR levels	Liang H et al (55)
		A549	Concentration: 1.7%, 3.4%, and 5.1%; Time: 2, 4, 6 h	Inhibition of cell proliferation, induction of apoptosis, and block of cell cycle progression	XIAP↓, suvivin↓, activating caspase-3, cyclin A↓, cyclin B1↓, cdc2↓	Liang H et al. (51)
		A549	Concentration: 1.5%, 2.5%, 3.5%; Time: 4 h	Suppression of hypoxia-induced growth and metastasis	HIF-1α↓, XIAP↓, survivin↓, fascin↓, HPA↓, p38 MAPK activity↑	Liang H et al. (60)
		A549	Concentration: 3% Time: 30 min	Increase of cell apoptosis	miRNA-155↓ miRNA-146a↑ miRNA-223↑	Wang L et al. (39)
	Glioma	U251; U87	Concentration: 4.1% Time: 4 h	Inhibition of cell proliferation, invasion and migration	miR-124-3p↑ suppression of ROCK1 signaling pathway	Gao C et al. (42)
	HNSCC	CAL-27; FaDu	Concentration: 2%, 4% Time: 2,4,6,8 h	Inhibition of cell proliferation, invasion and migration, and induction of cell apoptosis	Anti-proliferative effect: p-Akt↓ Cell apoptosis: activation of HIF-1α which regulates Fas/FasL signaling pathway	Yang YQ et al. (53)
	TSCC	SCC-4	Concentration: 4.1% Time: 24,72 h	Inhibition of tumor angiogenesis	Attenuate the hypoxia-induced VEGF level *via* increasing the DNA methylation	Lu Y et al. (37)
	Mouse colon carcinoma	MC-38	Concentration: 2.2% Time:45 min	Reduction in the invasion of CRCs	Impairment of neutrophil MMP-9 release and interference with pathways downstream of CXCR2, but upstream of PKC	Muller-Edenborn, B et al. (48)
	Breast cancer	MDA-MB; MCF-7	Concentration: 2% Time: 6 h	Suppression of cell proliferation	miR-203↑	Liu JY et al. (32)
	Colonic cancer	Caco-2	Concentration: 3% Time: 2 h	Induction of late apoptosis	Induction of p53- dependent apoptosis	S. KVOLIK et al. (52)
Desflurane	Mouse colon carcinoma	MC-38	Concentration: 6% Time: 45 min	Reduction in the invasion of CRCs	Impairment of neutrophil MMP-9 release and interference with pathways downstream of CXCR2, but upstream of PKC	Muller-Edenborn, B et al. (48)
	Epithelial ovarian cancer	Not mentioned	Clinical concentration	Improved DFS	Improved DFS	Elias KM et al. (61)
	Breast cancer	Not mentioned	Concentration: 3%–7%	Keeps the immune system stable	Preservation of CD4(+)/CD8(+) T cell ratio	
Xenon	Rat glioma	C6	Concentration:0.5–1 atm (0.5–1.6 MAC); Time:	Inhibition of physiological functions of the cancer cells	PMCA activity↓	Singh G et al. (67)
	Breast adenocarcinoma	MDA-MB-231; MCF-7	Concentration:70%; Time: 1, 3, 5 h	Inhibited cell migration and secretion of a pro-angiogenesis factor	RANTES↓	Ash SA et al. (66)
Isoflurane	Rat glioma	C6	Concentration:0.5%–4%; Time: 1–24 h	Enhancing glutamate uptake *via* increasing the expression and activity of EAAT3	Enhancing neurotoxicity in C6 cells *via* PKC– and PI3K–independent pathways	Huang Y et al. (68)
	Laryngeal papillomas	Laryngeal papillomas cells	Concentration: 1.4%; Time: 0.5 h	Inhibited cell proliferation and apoptosis evasion	Reduces COX2 enhancement and PGE_2_ release by inhibiting the activation of p38 MAPK	Ren HB et al. (70)
	Hepatic carcinoma	Hepatic carcinoma cells	Concentration:2 mg/ml; Time: 48 h	Inhibited cell growth and promoted cell apoptosis, inhibited cancer migration and invasion	Proapoptotic genes expression↑: capspase-3; caspase-8, anti-apoptotic mRNA expression↓: Bcl-2 and Bax; regulation of NF-κB activity and the PI3K/AKT signaling pathway	Hu J et al. (71)
Halothane	Mouse sarcoma	Mouse heteroploid strain; Mouse sarcoma I strain	Concentration: 0%–3.2%; Time: 4 days	Increase of glucose uptake and lactate output and inhibition of oxygen consumption	Mitochondrion	Fink B R et al. (75)
	Rat hepatoma	HTC	Concentration: 0.1%–5.0%; Time:24 h	Inhibition of cell multiplication and cell growth	Cell replication	Jackson S H, et al. (76)
	Rat hepatoma	HTC	Concentration: 0.1%–5.0%; Time:24 h	Inhibited the synthesis of DNA and cell replication	DNA synthesis	Jackson S H, et al. (77)
	Mouse neuroblastoma	N2A	Concentration:100 and 1000 ppm Time:4, 12, 24 h	Disruption of actin distribution; microspikes lost	Morphology changes	Uemura E, et al. (79)
	Breast cancer		Clinical halothane-nitrogen-oxygen concentration	Halothane anesthesia improved the survival rates of patients	Pituitary- adrenal cortex system; carcinemia development; tumor immunity	Fried I A (80)
	Melanoma	SK-MEL-37	Concentration: 4% Time: 3, 6, 12 or 24 h	Affect the progression of tumor cell metastasis	Tumor cell metastasis; ICAM-1	Azuma K et al. (83)
	Rat Glioma	C6	Concentration: 0.5%, 1%, 2%; Time: 8 min	Inhibited rat glioma cell growth	Morphology changes *via* RhoA, ERK, and Akt activation	
	Lung carcinoma	A549	Concentration: 1.5 and 2.1 mM; Time: 8 min	Reduction of viability; suppression of mitotic activity; disturbances of nuclear and nucleolar structures	Cellular structure changes	Stephanova, E et al. (81)
	Mouse neuroblastoma	NB2a	Concentration:0.3%–2.1%; Time: up to 72 h	Inhibited neuroblastoma cell differentiation and neurite extension; abolished microspike formation	Cellular morphological differentiation	HINKLEY R E et al. (82)
	Rat glioma cells; Rat neuroblastoma cells	C6;B104	Concentration:0.5%–1.75%; Time: 30 min	Might affect physiological functions of cancer cells	Inhibited PMCA activity	Singh, G et al. (67)
	Human colon carcinoma, larynx carcinoma, pancreatic carcinoma cells	Caco-2; HEp-2; MIA PaCa-2; SW-620	Concentration:1.5%; Time: 2, 4, 6 h	Growth suppression	Decrease in DNA, RNA and protein synthesis; DNA fragmentation	S. Kvolik et al. (78)

## Author Contributions

JW was in charge of the writing. C-sC was responsible for the pictures and editing. YL compiled the table and inserted the references. SS reconstructed and redesigned the work. SH made final agreement and approval of the work to be published. All authors contributed to the article and approved the submitted version.

## Conflict of Interest

The authors declare that the research was conducted in the absence of any commercial or financial relationships that could be construed as a potential conflict of interest.
